# The mysterious desert dwellers: *Coccidioides immitis and Coccidioides posadasii*, causative fungal agents of coccidioidomycosis

**DOI:** 10.1080/21505594.2019.1589363

**Published:** 2019-03-22

**Authors:** Daniel R. Kollath, Karis J. Miller, Bridget M. Barker

**Affiliations:** Pathogen and Microbiome Institute, Northern Arizona University, Flagstaff, AZ, USA

**Keywords:** *Coccidioides immitis*, *Coccidioides posadasii*, onygenales, comparative genomics, fungal pathogen

## Abstract

The genus *Coccidioides* consists of two species: *C. immitis* and *C. posadasii*. Prior to 2000, all disease was thought to be caused by a single species, *C. immitis*. The organism grows in arid to semiarid alkaline soils throughout western North America and into Central and South America. Regions in the United States, with highest prevalence of disease, include California, Arizona, and Texas. The Mexican states of Baja California, Coahuila, Sonora, and Neuvo Leon currently have the highest skin test positive results. Central America contains isolated endemic areas in Guatemala and Honduras. South America has isolated regions of high endemicity including areas of Colombia, Venezuela, Argentina, Paraguay, and Brazil. Although approximately 15,000 cases per year are reported in the United States, actual disease burden is estimated to be in the hundreds of thousands, as only California and Arizona have dedicated public health outreach, and report and track disease reliably. In this review, we survey genomics, epidemiology, ecology, and summarize aspects of disease, diagnosis, prevention, and treatment.

## Introduction

**Figure 1.**
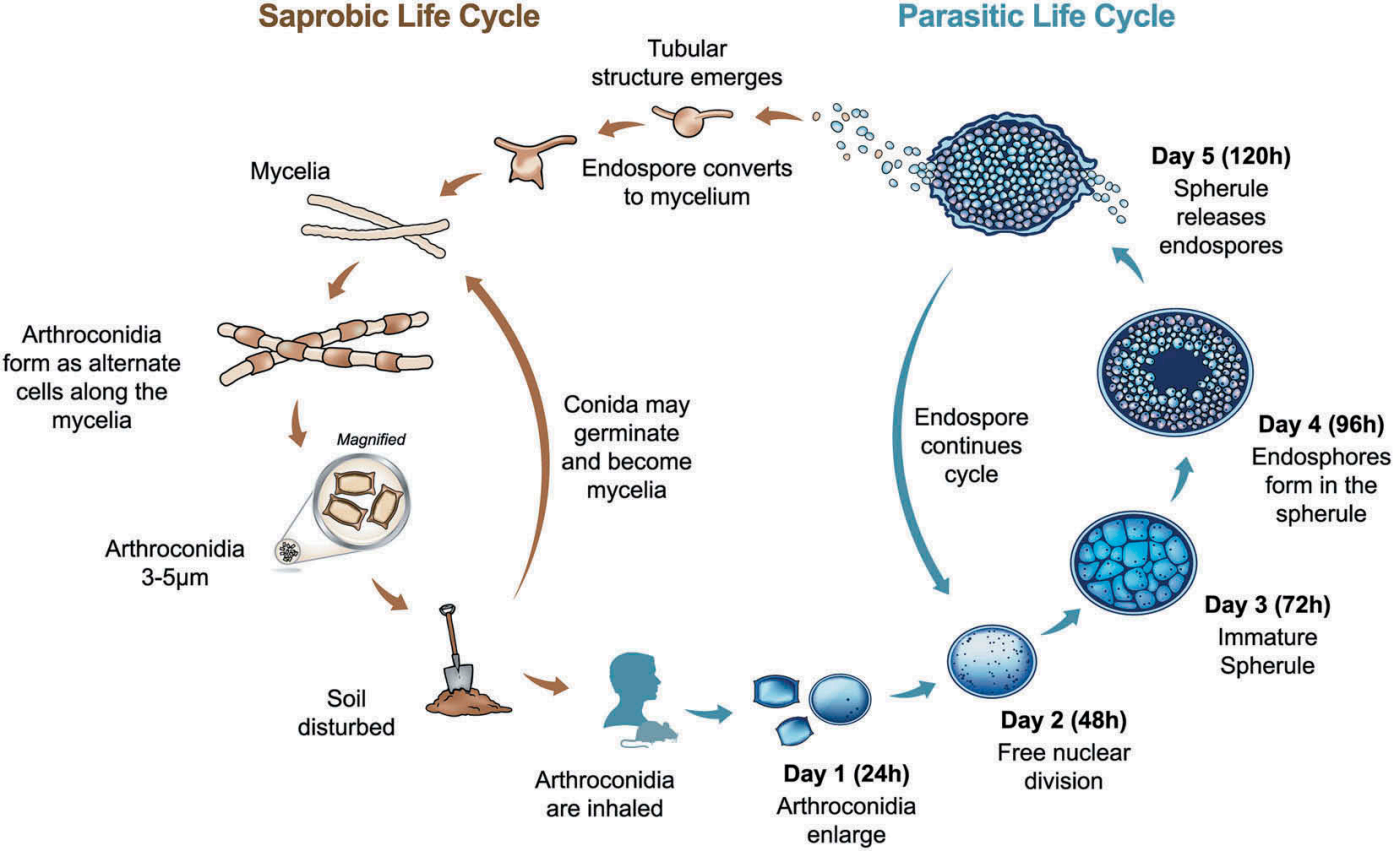
Life cycle of *Coccidioides *spp. During the saprobic phase (left) the organism grows as mycelia, which mature into arthroconidia. These asexual conidia can be inhaled by a susceptible host. If this occurs, the fungus undergoes a morphological shift to form a spherule (right). The spherule structure matures to contain endospores, which can potentially disseminate to other body sites in the host including skin, bones, or central nervous system.

The disease coccidioidomycosis, which is commonly known as valley fever (VF), was first described in the late 1800s in Argentina by Dr Alejandro Posadas [1]. The causative agent was first thought to be a protozoan that caused severe disease (thus, the etymology of *Coccidioides immitis*: Coccidia protozoan and immitis “not mild”) but was later identified as a dimorphic fungus, with most disease being asymptomatic or mild [2–4]. The unusual life cycle is defined by the large pathogenic structure called a spherule (Figure 1). The initial spherule develops from an inhaled arthroconidia, which is the asexual propagule that develops in the environment. This environmental life stage consists of nondescript mycelia that mature into alternating arthroconidia as the fungus grows and ages. The exact conditions required for growth and maturation in the environment are unknown, but evidence suggests that keratin sources and precipitation play a role [5,6]. Once infection is established, the spherule life stage predominates in the host, with endospores developing internally and the outer cell wall rupturing to release mature endospores. Each endospore can develop into a new spherule, and endospores are likely recognized and engulfed by host immune cells [7–9]. Once the spherule matures and enlarges, it cannot be engulfed and can rupture the host cell. Thus, it appears the *Coccidioides* can be both an intracellular and extracellular pathogen. The sexual stage of the life cycle has yet to be discovered, but appears to occur with high frequency [10,11].

Both organisms grow in arid to semiarid alkaline thermic soils throughout western North America and into Central and South America [12]. Endemic regions in the United States with highest predicted prevalence of disease include the Central Valley of California, southern Arizona, southwestern Texas, but only Arizona and California track and report disease prevalence to the Centers for Disease Control and Prevention (CDC), along with about half of the US states. The Mexican states of Baja California, Coahuila, Sonora, and Neuvo Leon currently have the highest skin test positive results, although Mexico no longer tracks the disease (pers comm Laura Rosio Castañón). Isolated areas in Guatemala (Motagua Valley) and Honduras (Comayagua Valley) have documented cases [13]. South America has several geographically isolated regions of endemicity including the northeastern area of Colombia; Lara and Falcon states in Venezuela; the Chaco region in Argentina/Paraguay; and Piaui, Maranhao, Ceara, and Bahai states of Brazil [14,15]. Disease prevalence in South America is not well characterized, possibly due to lower population densities and lower socioeconomic status in the regions of endemicity, but could also reflect the genotypes and phenotypes of both the pathogen and host found specifically in South America.

The genus *Coccidioides* consists of two species: *C. immitis* and *C. posadasii*. Prior to 2000, all disease was thought to be caused by a single species, *C. immitis*. However, genetic analysis clearly supports two distinct species [16]. Within each species, several populations have been proposed. For *C. immitis,* there are indications of population structure within the Central Valley, southern California/Baja California, Mexico, and a separate population in the newly identified endemic region of eastern Washington State [17–19]. For *C. posadasii,* a clear separation between isolates from Arizona and isolates from Mexico/Texas/South America has been consistently observed [19]. Additionally, Arizona isolates from the Phoenix region and the higher elevation Tucson region may be distinct subpopulations [17,18]. The true population structure will remain an enigma until direct isolations from soil are made throughout the range of the organism. Despite the clear genotypic variation, no clinical differences have been defined among species or populations – although no published reports have ever assessed phenotypic variation in this context.

The disease caused by *Coccidioides* is highly variable among human patients. The majority (60%) of patients are asymptomatic after infection [20]. For the remainder, symptoms can be mild, including pneumonia, and these infections normally resolve without intervention. However, if the infection become extrapulmonary, medical intervention may be necessary. Infections can disseminate to spleen, liver, brain, bone, and many other tissues in the body. Complications in diagnosis, treatment failure, and unusual presentations may result in severe disease progression, and even death. No vaccine for this disease is available, although efforts are underway to develop an effective vaccine. In this review, we summarize historical and recent developments in the study of *Coccidioides* and coccidioidomycosis.

## The ecology of *Coccidioides* spp

Most ascomycete fungi are saprotrophic in the environment and have an association with plants, but *Coccidioides* spp. has evolved the ability to infect immunocompetent mammals including humans [21]. Despite a dramatic increase in patients diagnosed with coccidioidomycosis in recent years, the ecology of the organism is poorly understood. Arid and semiarid soils of the southwestern United States, Mexico, Central, and South American are the natural reservoir for the fungus [12]. The distribution of the fungus in soil is inconsistent and unpredictable even in the endemic region where there is high disease burden [22]. There is evidence of an association between *Coccidioides* and animals (small desert mammals) due to greater detection of the fungus in close proximity to animal activity [23–28]. There is also evidence of climatic and seasonal variables that may influence growth and dispersion of the fungus leading to patterns of disease outbreaks [29]. This section will synthesize the information known about the ecology of *Coccidioides* spp. and propose areas for future research.

## Biotic factors: Soil

During the mycelial life cycle, *Coccidioides* spp. are thought to be a saprotrophic soil-dwelling fungi, although a preferred nutrient source is not described. The distribution in the soil is sporadic and irregular and may be driven by abiotic soil factors such as pH and electrical conductivity or possibly driven by biotic associations with small desert mammals such as rodents [27]. Few studies have tried to identify the abiotic soil variables that may be driving the distribution of *Coccidioides* spp. with limited support for any one predictor variable. In the 1970s, Lacy and Swatek investigated *C. immitis* around California archeological sites and reveled that sandy-textured soils made up of 98% of positive samples and 96.7% of positive soils were alkaline [30]. Elconin et al. also found a positive correlation between *C. immitis* isolation and increased soil salinity [31]. Though these studies provided evidence for an association with *C. immitis* and soils with alkaline pH, the samples sizes were quite small and relied on culture-based methods for fungal identification. Fisher et al. analyzed more abiotic variables, such as soil temperature, soil texture, chemical characteristics, and water quantity, which could affect the distribution and growth of *Coccidioides* throughout the southwestern United States [12]. The authors proposed that soils with low water content (water table is not near the surface) are more favorable for fungi, because as the soil dries microorganisms that can grow as filamentous hyphae may reach water pockets. Based on their assessment of laboratory and field studies, the optimal soil temperature range that promotes peak growth of the fungus is between 20 and 40°C and the soil texture (proportion of sand, silts, and clays in a given soil) in which *Coccidioides* is most commonly found is sandy loam (low water-holding capacity) with pH ranging from 6.1 to 8 with relatively low electrical conductivity. These data suggest that pH and texture are not limiting factors for the growth, but that temperature and water availability may be more important. The relatively few studies that have examined the role of abiotic factors do not provide strong evidentiary support for soil variables that can be used to predict the growth pattern and distribution of *Coccidioides* spp. in the environment [27].

## Environmental detection

Detection of the pathogen in soil is a difficult task as culture methods are shown to be insensitive (thousands of soils with no/few cultured strains) and mouse inoculation is expensive and time consuming with variable results [27,28,32–34]. With the development of new molecular technologies, it is easier and faster to detect presence of the pathogen using PCR, DNA sequencing, and real-time qPCR methods. Several methods target regions of the ITS or rDNA, and often require additional sequencing to verify the target identity [27,35,36]. A real-time PCR method targets a novel repetitive sequence that was first identified for use in a clinical diagnostic system [37,38]. The main benefit to molecular-based methods is the large number of soil samples that can be screened in a relatively short amount of time.

## Wind, dust, and airborne conidia

The role of dust in the dispersion of *Coccidioides* spp. propagules has been posited for many years, but has not been experimentally validated because has never been isolated from ambient dust and molecular detection is difficult [39–48]. Chow et al. were able to detect airborne *Coccidioides* in a simulated dust storm with relative success, but detecting the fungus in actual dust storms is much more difficult [41]. Climate models show a drying trend in the endemic areas for *Coccidioides* that increases the likelihood for dust storms [49,50]. Tong et al. showed that dust storms have increased 240%, from 1990s to the 2000s, in the southwestern United Stated and have a positive correlation with dust storm frequency and reported cases of coccidioidomycosis [51]. It is proposed that with the increasing frequency of dust storms comes a higher risk of inhaling infectious propagules. With the increase in dust and wind activity comes a greater need for better air surveillance techniques. After the California Northridge Earthquake in 1994, there was an outbreak of coccidioidomycosis that included three deaths. This outbreak was attributed to many landslides that generated massive dust clouds that blew into nearby densely populated valleys [44]. Stochastic events that can generate a large bolus of dust containing infectious propagules, such as earthquakes, may increase the possibility of outbreaks. There is also a risk for wind to disperse the fungus to “nonendemic” areas [40,42,44]. Weil et al. showed that dust storms can transplant entire microbial communities hundreds of kilometers (Saharan desert to the Italian Alps) including pathogenic “black-mold,” and these communities have the potential to become established in new areas [52]. Blowing dust has the potential to disperse infectious *Coccidioides* propagules to nonendemic areas, and should be monitored.

## Animal associations

Most fungi cannot survive higher temperatures and acidic pH of the mammalian body. Enduring the unforgiving conditions of desert soil microenvironments, such as extreme temperatures, dramatic pH shifts, and microbial competition via secondary metabolites may have led to promotion pathogenesis and the ability to infect mammals via “ready-made” virulence factors [53,54].

Although an animal reservoir has not been identified for *Coccidioides* spp., there is strong evidence of mammalian associations with pathogenic and nonpathogenic relatives. *Paracoccidioides brasiliensis*, a close pathogenic relative of *Coccidioides*, has been isolated from the feces of bats (*Artibeus lituratus*) and from the internal organs of the nine-banded armadillo [55,56]. There is evidence of animal association with another close pathogenic cousin of *Coccidioides, Blastomyces spp*. The fungus has been isolated from the feces of bats and from other various animal manures as well as from beaver dams [57–59]. There also is a strong association with prairie dog burrows which, like many other burrowing mammals, create designated latrine areas to store their waste that the fungus seems to prefer [60]. There is indication that fungal pathogens within the order Onygenales are associated with wild animals, either *in vivo* or *in situ*, and this close relationship may be an indication of how they evolved to become pathogenic in humans and other animals.

*Coccidioides* spp. as well as other fungi in the order Onygenales have the ability to degrade keratin and utilize it as a source of carbon, nitrogen, phosphorus, sulfur, amino acids, and other minerals [61,62]. The *Coccidioides* genome has a significantly reduced fungal-cellulose binding domain gene family that gives the fungus the ability to break down plant material suggesting that *Coccidioides* has reduced this capability [21]. The subtilisin N domain-containing family is highly expanded in the *Coccidioides* genome as well as in close relatives. This gene family contains the peptidase S8 family domain that encodes several keratinolytic subtilases (keratinases) and this gene family is three times larger in *Coccidioides* than in other taxa [21]. This genomic information suggests that unlike other fungal taxa in the sister order Eurotiales, which are often associated with plants and plant materials, *Coccidioides* and other Onygenales utilize animal-derived substrates, and may have lost ability to thrive on a vegetarian diet.

The ability to metabolize animal derived material may restrict where the fungus is growing in the environment. There is a large amount of animal material, such as keratin, in desert rodent burrows suggesting a suitable habitat for *Coccidioides*. Multiple studies have shown that most soils containing the pathogen are extracted from or in the vicinity to rodent burrows, and infected animals buried in soil can establish and grow [27,34,63]. In a recent study from the endemic area in Mexico, 82% of soils containing *Coccidioides* were taken from rodent burrows indicating a strong correlation to the burrow microhabitat [64]. The abundance of desert rodents inhabiting the endemic region of the fungus suggests a possible connection. In early studies *Coccidioides* was isolated from deer mice, pocket mice, ground squirrels, grasshopper mice, kangaroo rats, and pack rats [34]. Although these early studies were culture and morphology based, they provide evidence that desert rodents could be natural reservoirs that harbor and disperse the fungus in the environment. However, there is recent evidence that *Coccidioides* spp. is harbored in nonrodent animals such as bats and armadillos, and in some cases animals in captivity such as otters, kangaroos, and nonhuman primates have developed severe disease and had the fungus isolated from tissue, so specific reservoirs reamin to be defined [65–68].

This association differs from an opportunistic fungal pathogen like *Aspergillus fumigatus* which is an environmental saprobe that releases a large quantity of conidia into the air [69] *A. fumigatus* is a ubiquitous environmental fungus that is usually associated with plants, decaying organic material, marine and aquatic systems that typically infect humans when they are immunocompromised [70]. Animals are constantly being exposed to *Aspergillus* conidia, estimated few hundred per day, which does not lead to disease unless the immune system is compromised [71,72].

## Climate/seasonality

Changes in the environment can influence dispersal patterns of arthroconidia into the atmosphere that can lead to fluctuations in reported cases of VF [73]. There may be an association with increased incidence of reported disease with precipitation patterns based on the life cycle of the fungus. It is hypothesized that *Coccidioides* responds to soil moisture, so that when moisture is abundant the fungus grows as mycelium in soil and when the soil dries out specific viable hyphal cells mature into arthroconidia, which are released into the air [5,22,29,74–76]. This is the time when humans are at greater risk for inhaling infectious coccidioidal propagules. In Arizona, low precipitation in early summer correlates to higher incidences of coccidioidomycosis in the later summer (July, August, and September); but, when there is increased monsoonal activity in the early summer, there are lower incidences of VF in the later summer [22]. The authors show that there is also a positive correlation of increased incidence when there are high precipitation levels in the winter and spring months, and increased cases of VF in the summer months after heavy winter rains. These saturation events may give the fungus enough moisture to proliferate and create greater fungal biomass in the soil and when the soil dries out release more spores into the air. A complicating factor for all climate models to date is the reliance on human case report data, which may be months after the exposure event.

Temperature is another variable that may influence the growth of arthroconidia and lead to changed patterns of incidence in the endemic region. There is a hypothesis that the soil becomes sterilized by extreme high temperatures but *Coccidioides* survives by growing into deeper soil horizons, and then when there is rain the fungus can grow back to the soil surface [77]. Recent studies have shown that annual mean surface temperature is a significant driver of coccidioidomycosis cases. No counties in the endemic area have a mean surface temperature lower than 10°C and incidence rates higher than six cases per 100,000 people, whereas the counties with the highest incidence rate in California and Arizona (70 cases per 100,000 people) have a mean temperature that is greater than 16°C [49]. This suggests that temperature may be an important predictor variable of where the fungus prefers to grow in the environment.

The changing climate may create suitable habitat for *Coccidioides* spp. outside of the endemic region, although it is clear that there are areas of transient endemicity that suggest that the true area is larger than proposed [78]. Temperatures in the southwestern United States are expected to rise by 2°C, with the greatest increases expected during the summer and autumn months [79]. Previous work indicates that *Coccidioides* may prefer to grow in areas with higher surface temperatures; therefore, this warming trend might shift the endemic regions farther north into areas that may not have been suitable environment for the fungus before [49]. Drought projections show an intensification of drought throughout the current endemic area which can lead increased dust and dust events that will increase the rates of VF cases [49,80]. We propose that the changing climate may allow the pathogen to occupy new areas, expose more naïve hosts to the disease, and increase disease burden in already established endemic regions.

## Vaccines

A vaccine for VF was proposed by several groups [81–83]. It was observed that a primary infection seemed to protect individuals from subsequent infection, and most (60%) infections are not symptomatic [20]. Early vaccine tests were first assessed in guinea pigs, but without success [82]. After these frustrating starts, effective vaccines were developed for use in mice, monkeys, and dogs [84–86]. These early vaccine candidates relied on inactive whole cell and fungal components, both from the parasitic (spherule) phase and well as the environmental phase (conidia/mycelia) [87]. In fact, a formalin killed spherule vaccine went as far as Phase 3 human clinical trials. Nearly 3000 people received the vaccination, and 18 vaccinated individuals developed or were suspected to have developed mild VF, whereas 25 unvaccinated individuals developed or were suspected to have developed mild VF [88]. Based on these results, work aggressively shifted to development of specific antigen vaccines and attenuated strains. One of the most promising antigen-based vaccines was based on the development of a recombinant protein of antigen2 and a proline-rich antigen (Ag2/PRA), and the *Coccidioides* specific antigen (CSA) [89–92]. However, protection was still only 50–60% of mice surviving a challenge of ~200 conidia.

Single and multiple gene deletions in *Coccidioides* have resulted in attenuation or abolition of virulence. Some of these avirulent strains have been proposed to be used as a vaccine. In particular, the deletion of 2 chitinase genes was shown to protect a very susceptible mouse model; however, T-cell based immune response was indicated as critical for protection, and the authors suggested that the vaccine would be less effective in HIV/AIDS patients [93]. A current live attenuated vaccine is in development, and has shown highly protection in a mouse model of VF [94,95].

## Epidemiology

Steady increases in reported VF have been observed in the United States as regular reporting began in the 1990s [96,97]. In general, reported VF cases are highest in specific regions, primarily southern Arizona and the Central Valley of California. However, it is important to note that VF is only reported nationally in the United States, and is reported by only 24 states (and District of Colombia), and surprisingly known and suspected endemic states do not report disease including Texas, Oklahoma, Washington, Colorado, and Idaho, according to the CDC Morbidity and Mortality Weekly Report (https://www.cdc.gov/mmwr/index.html). No other countries in the endemic regions nationally report this disease, and therefore beyond the United States, there is not reliable data to understand if these increases are universal.

Delayed type hypersensitivity skin testing was used in early epidemiological surveys to determine regions of endemicity [98,99]. It was also used to determine the rate of infection among military personnel in California, and the first antigen used was called coccidioidin, which was administered intradermally [48]. Antigens derived from spherules (spherulin) rather than mycelia seemed to improve the sensitivity of the reaction, but not all confirmed VF cases reveal a positive skin test [100]. Importantly, patients with erythema nodosum should not receive the skin test due to potential tissue necrosis at the site of injection. Skin testing for determination of prior exposure may be useful to ascertain risk for certain occupations or among prison populations. Additionally, new epidemiological studies in novel endemic regions, such as eastern Washington State, are warranted.

Observed increases in disease could be attributed to improved reporting, diagnosis, and awareness [101]. Alternatively, these increases could be the result of changing climate, increased construction, and soil disturbance, as discussed above [5,36,40,73,102–104]. Predictions on the effect of changing climate on the incidence of VF in the southwestern US suggest that disease incidence will increase in endemic regions under warming and changing precipitation patterns [49]. The potential for expansion of the endemic region is a concern, and greater efforts regarding awareness of disease among clinicians and public health officials is critical for improving diagnosis and reporting

Certain patient populations have been shown to be at greater risk for severe disease. African Americans, Filipinos, pregnant women, and those with immunosuppressed conditions are well-documented groups at higher risk for dissemination [105–115]. Occupational exposures, such as working in construction, farm work, outdoor filing, solar farms, or archeological digs, have been associated with larger outbreaks among these workers [43,116–122]. Additionally, prison inmates and guards/workers in the endemic regions have high rates of exposure and disease [106,107,123–126].

There have been no studies that conclude that canines are more susceptible to coccidioidomycosis compared to humans, but infection may be more prevalent in canines due to behavioral tendency to disrupt soil [127]. As stated previously, infection is asymptomatic in 60% of human hosts and these rates in canines are comparable [128]. Early symptoms for both species include coughing, fever, weight loss, lack of appetite, and lack of energy. Most commonly, canines may not express symptoms of a lung infection but at the minimum, will show signs of an active disseminated disease such as lameness and seizures, which will allow early detection of valley fever. Due to the ambiguity of the symptoms, diagnosis depends on specific tests (summarized below) in addition to the clinical symptoms.

## Diagnosis

Several methods have been developed to diagnose VF. In addition to clinical diagnosis of symptoms, direct culture or histopathological evidence of the organism, and radiographic findings; diagnostics that have been used include tube precipitin (TP), complement fixation (CF), immunodiffusion, agar gel precipitin-inhibition, latex particle agglutination (LPA), and enzyme-linked immunosorbent assays (ELISA). There has been recent work suggesting the use of a peptide microarray based on immunosignatures to diagnose VF. These proposed peptide diagnostics were shown to be extremely sensitive, and cross react with other related infections [129]. Charles Smith and colleagues developed the TP and CF tests in the 1950s [130,131]. Interestingly, the authors observed that TP positive reactions occurred within weeks of infection, whereas CF positivity occurred 2–3 months after infection, and CF titers could increase if infection was not controlled. In 2015, a new delayed-type hypersensitivity skin test was development and showed promise as a noninvasive diagnostic for VF with no cross-reactivity to other related infections such as histoplasmosis; but may also miss positive reactors and underestimate disease [132]. It is now known that this reflects immunoglobulin M (IgM/TP) and immunoglobulin G (IgG/CF). IgM-positive reactions likely occur in the first few weeks of illness, whereas the IgG reaction becomes positive later in disease, and titers may increase if infection is uncontrolled [133]. Similar to TP, LPA testing detects primarily IgM [134]. In asymptomatic cases, IgM and/or IgG may be detected, but titers may become nondetectable after the resolution of infection. A serological ELISA method based on the detection of both IgM and IgG show high specificity and sensitivity, 98.5 and 95.5%, respectively, and is commonly used for diagnostics [135].

Some studies have discussed difficulties in antibody detection during early time-points of the infection, as well as in immunosuppressed patients [136]. An alternative approach is the detection of fungal antigens in biofluids (typically sera) via antigen enzyme immunoassay [137]. For example, antibodies against fungal galactomannan could improve detection of coccidioidomycosis [138]. Cross-reactivity with other mycosis was shown in this report, so multiple diagnostic tests may be required and interpretation of results should consider the possibility of infection with other etiologic agents.

Molecular assays have been developed starting in the 90s, based on DNA hybridization and PCR/qPCR based methods, some mentioned above have been used in both clinical and environmental detection schemes. Detection and genotyping commonly relies on sequencing rDNA (both 18S and ITS1-5.8S-ITS2 have been targets). The ITS2 region in particular can be targeted for species specific detection [139]. A rapid and specific real-time qPCR that uses a Taq-Man probe to target a unique LTR retrotransposon detected both *C. posadasii* and *C. immitis* and is commercially available [38,140]. Many fungal genomes have been sequenced to date, and are available for development of sophisticated molecular tools to detect *Coccidioides* biomarkers [17,141].

## Antifungal drugs/treatment

Antifungal treatment recommendations for coccidioidomycosis are dependent on the clinical severity. The duration of treatment can range from 3–12 months to lifelong treatment. The deadliest infections include meningitis or the dissemination to the central nervous system and it is recommended that these cases are given lifelong antifungal medication [142]. Studies investigating the effects of ceasing azole therapy for *C*. *immitis* have further demonstrated that with any level of infection, long-term azole or triazole therapy is suggested to prevent relapse that could lead to a more serious *Coccidioides* infection, especially if one is immunocompromised [143].

Amphotericin B was introduced in the 1950s and quickly became the antifungal drug of choice due to its efficiency in clearing systemic fungal infections [144]. Although its use as an antifungal drug was life-saving, the nephrotoxicity was underestimated and renal failure, mortality rate, and additional financial costs were not trivial [145]. This drug became a “Gold Standard” but was only used when other antifungal treatments failed due to side effects. This led to the development of other antifungal drugs for the treatment of VF, such as fluconazole in the early 90s that had fewer side effects and toxicity [146,147].

The most common class of antifungal treatments for VF are the azoles that target ergosterol biosynthesis, and the polyenes that bind ergosterol. Ergosterol is a component of the fungal cell membrane, and these drugs often cease fungal growth but are not fungicidal. Common drugs that are administered for the treatment of coccidioidomycosis are fluconazole, itraconazole (azoles), and amphotericin B (polyene) [148]. Both species of *Coccidioides* have shown variable resistance to the listed antifungal medications [149–151]. Further work is needed to determine the mechanism of fluconazole resistance in *Coccidioides*. It is currently unclear whether emerging resistance is concerning in a clinical setting, and the conditions under which antifungal resistance needs to be monitored.

## Conclusions

Coccidioidomycosis is a potentially severe and understudied fungal infection. The regions of endemicity are often associated with lower socioeconomic status, and infections may be exacerbated by health disparities and other comorbidities. Both species are found in association with animals in the desert environment, but a lack of specific knowledge of the ecology and effects of climate make prediction of the future risk of increase in disease with climate change complicated. Diagnostics are imprecise and often complicated if the host is immunosuppressed. No vaccine exists and treatment is based on standard antifungal drugs.
